# Chitosan-based hydrogel to support the paracrine activity of mesenchymal stem cells in spinal cord injury treatment

**DOI:** 10.1038/s41598-019-42848-w

**Published:** 2019-04-25

**Authors:** M. Boido, M. Ghibaudi, P. Gentile, E. Favaro, R. Fusaro, C. Tonda-Turo

**Affiliations:** 1 Neuroscience Institute Cavalieri Ottolenghi, Department of Neuroscience, University of Torino, Orbassano, 10043 Italy; 2 School of Engineering, Newcastle University, Newcastle Upon Tyne, NE1 7RU United Kingdom; 3 Department of Medical Science, University of Turin, Torino, 10126 Italy; 4 Department of Mechanical and Aerospace Engineering - PolitoBIOMed Lab, Politecnico of Torino, Torino, 10129 Italy

**Keywords:** Biomedical engineering, Stem-cell therapies

## Abstract

Advanced therapies which combine cells with biomaterial-based carriers are recognized as an emerging and powerful method to treat challenging diseases, such as spinal cord injury (SCI). By enhancing transplanted cell survival and grafting, biomimetic hydrogels can be properly engineered to encapsulate cells and locate them at the injured site in a minimally invasive way. In this work, chitosan (CS) based hydrogels were developed to host mesenchymal stem cells (MSCs), since their paracrine action can therapeutically enhance the SC regeneration, limiting the formation of a glial scar and reducing cell death at the injured site. An injectable and highly permeable CS-based hydrogel was fabricated having a rapid gelation upon temperature increase from 0 to 37 °C. CS was selected as former material both for its high biocompatibility that guarantees the proper environment for MSCs survival and for its ability to provide anti-inflammatory and anti-oxidant cues. MSCs were mixed with the hydrogel solution prior to gelation. MSC viability was not affected by the CS hydrogel and encapsulated MSCs were able to release MSC-vesicles as well as to maintain their anti-oxidant features. Finally, preliminary *in vivo* tests on SCI mice revealed good handling of the CS solution loading MSCs during implantation and high encapsulated MSCs survival after 7 days.

## Introduction

A traumatic spinal cord injury (SCI) leads to permanent disability. Although advances made in the understanding of the pathogenesis and improvements in early diagnosis and treatment, approximately 2.5 million people worldwide^1^ live with the effects of SCI, which following functional deficits lead to disastrous social and human consequences and huge economic costs.

Particularly after SCI, a cascade of events alters the microenvironment at the trauma site^2^. During the acute phase, the outcome of the injury is unpredictable, and surgical approaches are directed to lesion stabilisation. An intervention at the intermediate phase, before the complete formation of glial scar (a physical obstacle to axonal regeneration), maximises treatment effectiveness and, at the same time, promotes tissue regeneration. When intervention occurs at the late phase, the process of glial scar formation has been already completed and axon regeneration and functional recovery become impossible thus far.

To date, successful treatment of SCI remains an open issue and innovative approaches are required to improve patient’s quality of life. This is due to the complexity of the anatomy of the spinal cord and the cascade of events occurring at the damage site^3^ that limits dramatically the treatment options which should be able to reduce glial scar formation as well as allow the neural regeneration integrating both neuroprotective and neuroregenerative cues^4^.

Several methods have been proposed to fabricate device for SCI treatment including (i) 3D porous scaffolds for gap bridging, and (ii) localized release of drugs, trophic factors and cells^5^.

Concerning 3D scaffolds, the influence of extracellular matrix (ECM) derived peptides on cellular response of neural cells have been reported, showing an effective increase of cell binding and neurite extension for RGD or YIGSR and IKVAV, respectively^3,6^. Furthermore, the importance of scaffold anisotropic structure has been studied: scaffolds characterized by structures having aligned fibres or channels in the direction of axon regeneration have been significantly improved the axon regeneration^6^ as well as the incorporation of chemotactic cues^3^ or cells^7^. However, these scaffolds failed the in human translation as required an invasive surgery to be implanted resulting in poor applicability in clinics.

On the other hand, the local release of drugs, trophic factors and cells offers the opportunity to localize specific cues at injury site through minimally invasive techniques reducing the risks to compromise an injured environment. A variety of neurotrophic factors have been applied in the treatment of SCI in the attempt to assure survival of damaged cells and to foster regrowth of injured axons^8^. Polymeric carriers mainly in form of hydrogel have been applied for the development of drug delivery systems; several proteins, such as neurotrophins and GDNF, have been incorporated and released at the injured site with finally reducing glial scar formation^9^. Although the local release of therapeutic factors has reported promising results, the short half-life of the factors and the difficulties to achieve prolonged released have compromised their application in clinics.

Current trends in the treatment of SCI are addressed to tackle localized factor release exploiting the ability of cells to release such factors by designing cell-based treatments able to be applied in a minimally invasive way. Mesenchymal stem cells (MSCs) have been proposed as a therapeutic tool thanks to their secretome (i.e. their ability to secrete a broad range of factors) having several beneficial effects as promoting neurogenesis, inhibiting apoptosis and glial scar formation, enhancing immunomodulation, angiogenesis, neuronal and glial cell survival, as well as relevant neuroprotective actions on different pathophysiological contexts^2,10,11^.

However, recent studies have revealed a limited survival time and low engraftment rate after cell implantation as implanted cells do not survive for long and the survival rate is strongly reduced when cells are implanted into an injured and highly reactive environment. Current options in cell-based therapies are addressing these challenges by applied hydrogels as cell carriers to enhance both cell survival and engraftment in the injured area. Several biomaterials have been processed to fabricate cell housing hydrogels for different tissue engineering applications^12–14^. In the contest of SCI, very few examples are reported in literature and in all cases inert polymers (which do no interact with the surrounding environment) were applied as former materials such as polyurethane-based polymers^15^ and poly(2-hydroxyethyl methacrylate)^16^. In this work, we proposed for the first time the use of chitosan (CS)-based hydrogels as a cell carrier for the treatment of spinal cord injuries through paracrine release of trophic factors from MSCs. Chitosan was selected not only for its well-documented biocompatibility^17^, easy processability, low immunogenicity but mainly for its anti-inflammatory and anti-oxidant properties^18^ which in combination with MSCs can orchestrate the inflammation process toward the establishment of a less hostile environment after traumatic diseases. In this work, physico-chemical properties of the CS-based hydrogel were analysed as well as its ability to encapsulate MSCs and to guarantee their survival and the release of trophic factors from encapsulated cells. Then, preliminary *in vivo* analyses on SCI mice were performed.

## Results

### pH values and gelation time at 37 °C of the CS/β-GP solutions

The pH and the gelation time of the tested CS/β-GP solutions are reported in Table 1. The pH of the initial CH solution was 5.8 ± 0.1 and the addition of different β-GP contents increased the pH values up to 7.11 ± 0.03 as well as reduced the gelation time up to 5 minutes A fast gelation and a physiological pH are the two key features to apply the CS/β-GP solution as cell carrier. Data reported in Table 1 suggests that the optimal CS/β-GP solution was obtained using a 2.5% (w/V) final CS/β-GP concentration with a ratio (R) between β-GP moles and the moles of amino groups into the CS chain of 4. This formulation has been used for all the following characterizations performed on the hydrogels.

**Table 1:** pH values and gelation times measured through the tube inversion method of different CS/β-GP solutions.

		Final CS/β-GP concentration (w/V)	
		2%	2.5%
R = 1	Gelation time (min)	410	250
	pH	6.67 ± 0.08	6.62 ± 0.03
R = 1.5	Gelation time (min)	73	60
	pH	6.83 ± 0.02	6.77 ± 0.02
R = 2	Gelation time (min)	35	20
	pH	6.85 ± 0.02	6.89 ± 0.04
R = 2.5	Gelation time (min)	27	15
	pH	7.02 ± 0.02	6.94 ± 0.05
R = 3	Gelation time (min)	20	10
	pH	7.00 ± 0.02	7.03 ± 0.02
R = 3.5	Gelation time (min)	14	7
	pH	7.08 ± 0.01	7.07 ± 0.03
R = 4	Gelation time (min)	12	**5**
	pH	7.16 ± 0.05	**7**.**11 ± 0**.**03**

The final % of CS and the amount of β-GP varied as reported.

### CS hydrogel morphology

**Figure 1:**
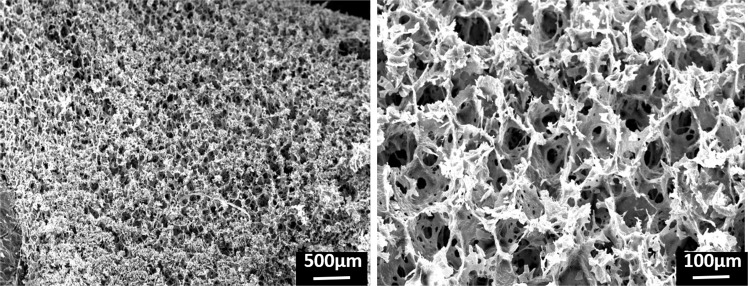
SEM micrographs of freeze-dried CS/β-GP hydrogel.

SEM analyses (Fig. 1) revealed a porous structure of the freeze-dried CS/β-GP hydrogel with a two-scale porosity: a macroporosity with pore size around 112 ± 23 µm and a microporosity with pore size around 15 ± 7 µm (measured through Image J). This high porosity is ascribed to the high amount of water in the CS/β-GP hydrogel theoretically estimated as around 86% of the total weight.

### Fourier transform infrared-attenuated total reflectance spectroscopy

**Figure 2:**
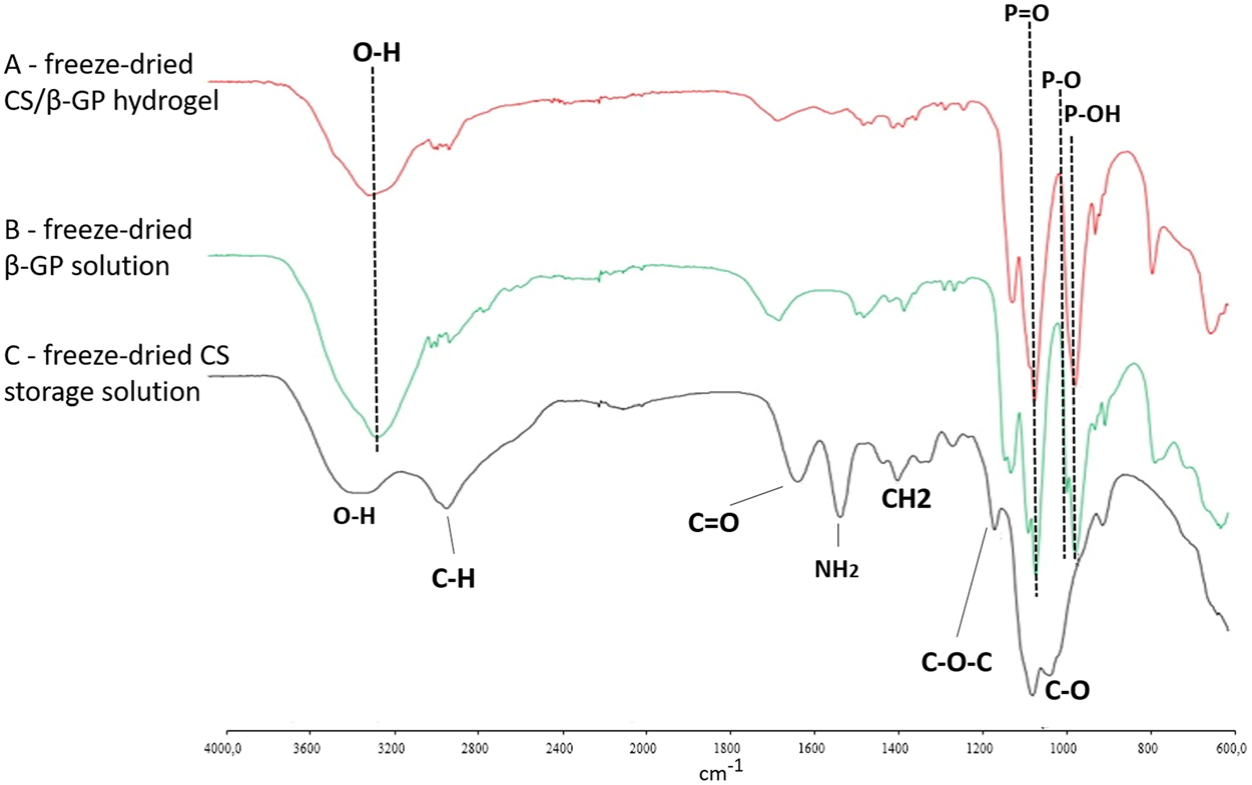
FTIR spectra of (**A**) freeze-dried CS/β-GP hydrogel, (**B**) freeze-dried β-GP solution and (**C**) freeze-dried CS storage solution.

Figure 2 reports the FTIR-ATR spectra of freeze-dried CS/β-GP hydrogel, β-GP and freeze-dried CS storage solution. The characteristic peaks of CS at 3281 cm^−1^ (*stretching* O-H), 2882 cm^−1^ (*bending*–CH), 1613 cm^−1^ (*stretching* C=O of secondary amine), 1513 cm^−1^ (*bending* -NH_2_, primary amine), 1379 cm^−1^ (*bending* -CH_2_, 1151 cm^−1^ (*stretching* C-O-C), 1062 cm^−1^ (*stretching* C-O)^19^ are present in the freeze-dried CS storage solution and freeze-dried CS/β-GP hydrogel (Fig. 2A,C). On the other hand, the peaks for β-GP were detected as 3202 cm^−1^ (-OH bond), 961 cm^−1^ (P-OH bond), 1112 cm^−1^ (P=O bond) and 1054 cm^−1^ (P-O bond)^20^ (Fig. 2B). CS/β-GP hydrogel spectrum (Fig. 2A) reveals a prevalence of peaks related to β-GP. This is in line with the composition of the CS/β-GP hydrogel where the amount of salt is 6-fold higher than the CS. Furthermore, no new peaks were detected in the β-GP hydrogel spectrum confirming the absence of chemical bond between CS and β-GP.

### Qualitative analysis of hydrogel injectability

CS/β-GP solution at 4 °C was successfully injected through the 4 needles (21 G, 22 G, 25 G, 30 G) using the CS/β-GP hydrogel using the different flow rates (3, 5, 8 and 10 mL h^−1^). When injected into water at 37 °C, CS/β-GP solution underwent a phase transition becoming a hydrogel and forming a stable wire (Fig. S1). On the other hand, no gelation occurred when the CS/β-GP solution was injected into water at 4 °C.

### Rheological tests

**Figure 3:**
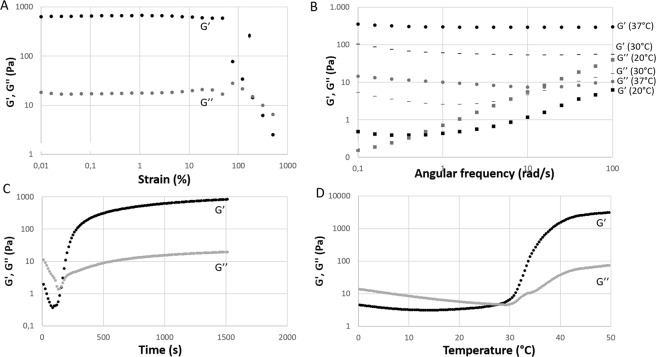
Representative curves of storage modulus (G’, black) and loss modulus (G”, grey) of CS/β-GP solution as a function of several parameters. (**A**) The shear strain test shows the behavior of G’ and G” as a function of the applied strain at 37 °C. (**B**) Frequency sweep tests reporting the frequency dependence of G’ and G” different temperatures (37 °C, dot – 30 °C, line and 20 °C, square). (**C**) The time sweep test measures G’ and G” values when temperature was instantaneously increased from 0° to 37 °C. (**D**) The temperature sweep test shows the effect of temperature on G’ and G”.

Values of storage modulus (G’) and loss modulus (G”) for different strain values are reported in Fig. 3A showing a linear behaviour up to a critical value of shear strain (around 80%) where an overshoot phenomenon occurred. A shear strain value in the linear region was chosen for the following rheological tests.

Frequency sweep tests were performed (Fig. 3B) at different temperatures (20 °C, 30 °C and 37 °C) with increasing angular frequency. At temperatures higher than room temperature (30° and 37 °C), CS/β-GP solution displayed gel behaviour as G’ is higher than G” and the gap between G’ and G” values is constant resulting in parallel trend lines. At 37 °C stronger gels were formed as the gap between G’ and G” was higher than at 30 °C confirming the importance of the temperature in the physical and mechanical properties of the CS/β-GP solution. For lower temperatures as 20 °C, CS/β-GP solution displayed a viscoelastic solid-like behaviour for low frequency where G’ is higher than G” and the gap among values depends from angular frequency while for higher frequencies the systems showed a viscoelastic liquid-like behaviour that is observed when G” is higher than G’.

The gelation process of CS/β-GP solution was monitored by means of the time sweep test at 37 °C and the temperature sweep test (Fig. 3C,D, respectively). The crossover point where G’ crosses and G” becoming higher than G” distinguished from sol state to gel state^21^ determining the CS/β-GP hydrogel formation. When the CS/β-GP solution was placed at 37 °C the gelation occurred after 144 ± 12 seconds (crossover point) reaching the complete gelation after 200 seconds as the gap among G’ and G” remained constant (Fig. 3C). Likewise, the transition from sol to gel state occurred at temperature 29 ± 1.5 °C as reported in Fig. 3D.

### Equilibrium water content and stability tests

**Figure 4:**
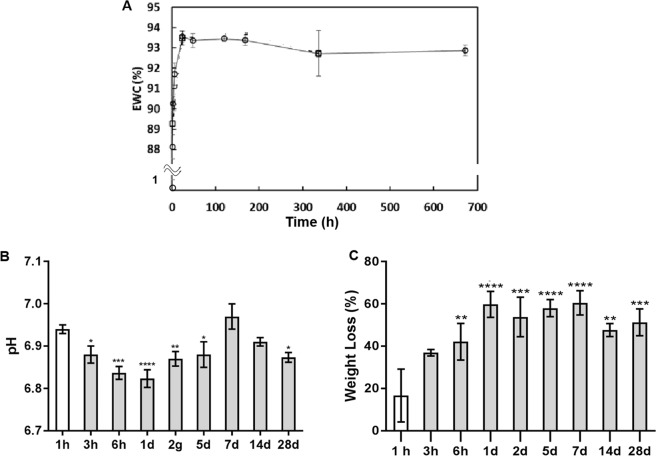
Equilibrium Water Content (EWC) of CS/β-GP hydrogel in PBS at different time points (**A**). Variation of PBS pH values (**B**) and weight loss (**C**) after CS/β-GP hydrogel immersion.

CS/β-GP hydrogel is a highly hydrated network characterized by a theoretical composition of 86% of water, 11.55% of β-GP, 1.95% of CS, 0.5% of HCl. When immersed in PBS solution, the amount of water increased reaching the maximum value (93.5%) after 2 days (Fig. 4A). Then, the amount of water within the hydrogel network remained stable up to 28 days. The stability of the CS/β-GP hydrogel in physiological environment was calculated by monitoring the weight loss in PBS. The weight loss started immediately after immersion in PBS and increased up to 1 day remaining constant until 28 days. This behaviour can be ascribed to the release of unbound β-GP after immersion of the hydrogel in PBS as CS was not detected into the supernatant through Ninhydrin assay. WL kinetic confirmed the formation of a stable network after gelation characterized by a hydrophilic network stabilized by hydrophobic domains^22^. The interaction among CS hydrophobic domains hindered CS/β-GP hydrogel dissolution and permit a long-lasting cell engraftment *in situ*.

Finally, the pH values in the supernatant were monitored at different time points reporting a slight reduction of the pH within the first days related to the exchange of salts and ions among hydrogel and PBS (pH range from 7.05 to 6.85).

### Permeability

**Figure 5:**
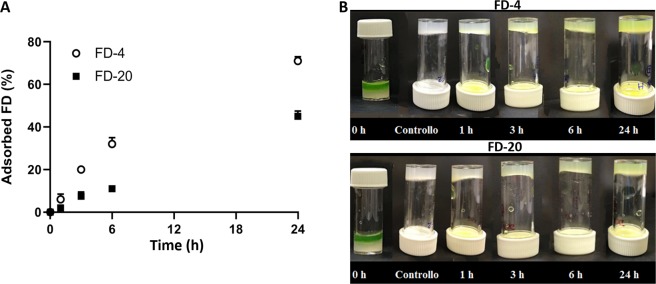
(**A**) Amount of FD-4 and FD-20 adsorbed by CS/β-GP hydrogel as a function of time. Percentage was calculated respect to the initial concentration of the solutions loading FD-4 and FD-20, respectively. (**B**) Representative images of FD-4 and FD-20 uptake. At time 0, a PBS solution containing the model molecules was added on the top of the CS/β-GP hydrogel. The change in color of CS/β-GP hydrogel was monitored within 24 hours. After 24 hours, the model molecules were uptaken by the CS/β-GP hydrogel for its whole thickness both using FD-4 and FD-20.

The permeability to nutrients and small proteins of the CS/β-GP hydrogel was assessed using two fluorescein isothiocyanate−dextrans having different Mw (FD-4 and FD-20). Figure 5 shows the adsorption kinetic of the two dextrans which was affected by dextran size as a faster adsorption was observed for the smaller molecule (FD-4) compared to FD-20. The reported data confirmed the higher permeability of the hydrogels as after 24 hours more than 40% and 70% of FD-4 and FD-20 content was adsorbed, respectively (Fig. 5A). FD-4 shows a higher diffusion coefficient (3,5E-10 m^2^ s^−1^) compared to FD-20 (1,4E-11 m^2^ s^−1^).

Moreover, the ability of the CS/β-GP hydrogel to uptake substances from the external media was confirmed by visual inspection (Fig. 5B). After 24 hours, the whole thickness of the hydrogel was yellow-coloured quantitatively confirming the permeability of the CS/β-GP to both FD-4 and FD-20.

### Preliminary observations of cell viability in presence of CS/β-GP hydrogel

**Figure 6:**
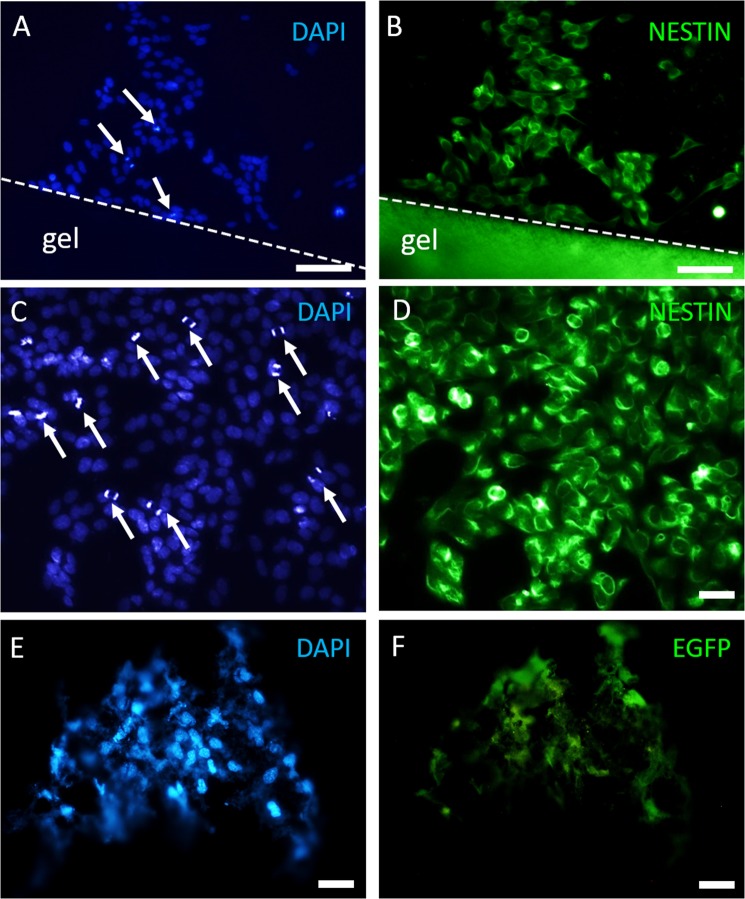
Cells grown on CS/β-GP hydrogel *in vitro* (7DIV). SH-SY5Y DAPI nuclei (**A**,**C**) with mitotic cells (white arrows in **A**,**C**) and cell cytoskeleton labelled by nestin (**B**,**D**) are visible near CS hydrogel (CS/β-GP hydrogel shows a high autofluorescence in the green channel). MSC DAPI nuclei (**E**) and EGFP MSCs (**F**) inside CS/β-GP hydrogel. Scale bars: (**A**,**B**) 100 µm; (**C**–**F**) 30 µm.

To evaluate the biocompatibility of CS/β-GP hydrogel, we first carried out a series of *in vitro* experiments using both MSCs and SH-SY5Y as these cell populations were used for hydrogel encapsulation and as a model of the neuronal cells of the spinal cord, respectively. SH-SY5Y cells were grown close by while EGFP + MSCs were grown inside a 100 µl drop of CS/β-GP hydrogel. At 7 DIV, surviving SH-SY5Y cells were found near the hydrogel as demonstrated by immunocytochemistry (DAPI- and Nestin-positivity; Fig. 6A–D). Moreover, several SHSY-5Y cells underwent mitoses near the hydrogel (arrows in Fig. 6A and, at higher magnification, in Fig. 6C), indicating that the presence of the CS hydrogel did not affect the survival and the cell division of the surrounding cells, allowing SH-SY5Y proliferation.

For MSC culture, EGFP-positive cells were used in order to be easily identified avoiding immunofluorescence reactions. MSCs were perfectly visible inside the hydrogel, as demonstrated by EGFP- and DAPI positivity (Fig. 6E,F). MSCs showed their typical fibroblast-like morphology, suggesting CS/β-GP hydrogel as a suitable biocompatible material for MSC encapsulation.

### Cytotoxicity assay: 3-(4,5-dimethylthiazol-2-yl)-2,5-diphenyltetrazolium bromide (MTT) assay

**Figure 7:**
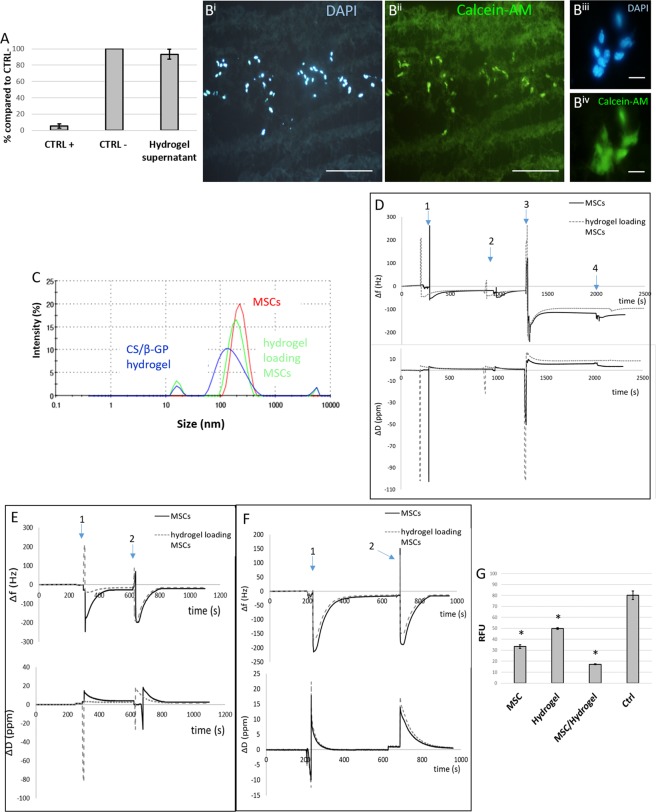
(**A**) MTT assay. The percentage of live cells was calculated for CTRL+ and MSC medium + CS, compared to CTRL− conditions. (**B**) Representative images of cells encapsulated in CS/β-GP hydrogel after 5 days (DAPI-blue and calcein-AM-green staining), at low (B^i^-B^ii^) and high magnification (B^iii^-B^iv^). (**C**) Detection of MVs released by MSC alone or encapsulated into CS/β-GP hydrogel through DLS analysis; (**D**) QCM-D plot of the seventh overtone of Δf and ΔD versus time using an Au sensor where event 1 was due to PEI solution pouring on AU sensor, event 2 to rinse of PEI coated sensor, event 3 to MV pellet suspended in PBS poured on crystal surface and event 4 to final rinsing step; (**E**) plot of the seventh overtone of Δf and ΔD versus time using an anti-CD9 coupled sensor where event 1 was due to MV pellet suspended in PBS poured on crystal surface and event 4 to final rinsing step; (**F**) plot of the seventh overtone of Δf and ΔD versus time using an anti-CD81 coupled sensor where event 1 was due to MV pellet suspended in PBS poured on crystal surface and event 4 to final rinsing step; (**G**) DCFHA-DA assay: the oxidative stress (expressed as RFU) induced in SH-SY5Y cells by H2O2 is reduced by adding MSC, CS/β-GP and MSC + CS/β-GP supernatant in comparison to CTRL + growth condition. Scale bar: (B^i^-B^ii^) 200 μm, (B^iii^-B^iv^) 20 um.

To further confirm the previous data concerning CS/β-GP biocompatibility, the MTT assay was performed using MSCs being the cells used for cell transplantation. Three different MSC culture conditions were compared: cells grown in (i) MSC medium (CTRL−); (ii) the supernatant collected from CS/β-GP hydrogel; (iii) MSC medium containing 30% DMSO (CTRL+). Only in live cells specific enzymes reduce the MTT dye to formazan crystals that make the solution of purple colour. As indicated in Fig. 7A, both “CTRL−” and “hydrogel supernatant” conditions allowed the release of MTT dye at a comparable percentage (100 ± 2% CTRL+ and 101.77 ± 7% MSC medium + CS), while no signal of live cells was measured for “CTRL−”.

Such results indicate a high biocompatibility of CS/β-GP hydrogel, since no significant differences were observed between MSCs cultured using normal media, and media containing CS/β-GP hydrogel supernatant.

### Viability of MSCs encapsulated into CS/β-GP hydrogel

To further exclude the CS/β-GP hydrogel cytotoxicity, we performed the calcein-AM/ethidium homodimer-1 assay. The metabolic conversion of non-fluorescent calcein-AM to fluorescent calcein allows to visualize a green signal in the live cells, whereas the binding of ethidium homodimer-1 to cell DNA (that becomes accessible in disrupted cell and nuclear membranes) produces a red fluorescence in the dead cells. The MSCs were loaded into the CS/β-GP hydrogel by gently mixing and the presence of cells did not affect the gelation time and temperature of the hydrogel as confirmed by visual inspection. The viability of MSCs was evaluated after 5 day-encapsulation into CS/β-GP hydrogel. Figure S2 clearly indicates that the viability of MSCs was unaffected by the presence of CS/β-GP hydrogel, as very rare red nuclei can be detected. Conversely the green signal was strong and evident: as also represented in Fig. 7B, MSCs showed a similar survival in presence of CS/β-GP compared to normal culture conditions. Figure 7B (Bi-Biv) displays a representative slice of the cryostat cutting MSC-loaded hydrogel stained by DAPI (nuclei) and calcein-AM (live cells). Indeed, at high magnification (Fig. 7Biii-Biv), the green signal appeared bright and intense, as well as the nuclei morphology was well preserved (no sign of apoptosis was visible).

Moreover, to assess the paracrine activity of MSCs in presence of CS/β-GP hydrogel, the DLS technique was employed for estimating the MV release and their respective size. The experiment was conducted comparing the content of the supernatant of MSCs grown either within CS/β-GP hydrogel or in standard conditions. In Fig. 7C, a sharp peak for 100–400 µm particle size was visible for both MSCs alone or encapsulated into CS/β-GP hydrogel. CS/β-GP hydrogel alone was used as control to confirm that the degradation product of the hydrogel did not have a size comparable to MSC-MVs. These results suggest that CS/β-GP hydrogel did not affect MSC ability to release MVs as indicated by the DLS curves almost overlapping (red and green lines in Fig. 7C).

Furthermore, the paracrine ability of MSCs, encapsulated or not into CS/β-GP hydrogel, was confirmed by quartz cristal microbalance with dissipation monitoring (QCM-D) measurements^23,24^. Au crystal coated with PEI was used to measure the presence of MVs in supernatants as the vesicles have a negative charge that allows the interaction with the PEI positively charged surface^25,26^. Δf plot confirmed the formation of a stable PEI coating (Fig. 7D event 1 and event 2) then MVs collected from supernatants of MSCs and CS/β-GP hydrogel loading MSCs were poured on PEI coated crystals. In both conditions, an increased in mass adsorbed on the PEI coated crystals was recorded (negative Δf in Fig. 7D event 3 and 4), confirming the presence of negatively charged MVs. Furthermore, specific antigens, able to label MVs^27,28^ (i.e. anti-CD9 and anti-CD81), were covalently immobilized on NHS-amine coupling sensors. Negative Δf values were measured in all conditions tested highlighting the presence of CD9 and CD81 positive MVs in the collected supernatants (Fig. 7E,F). In all the experiments, the amount of measured MVs was slightly higher for MSCs alone compared to MSCs loaded into the CS/β-GP hydrogel, since MSC-released vesicles have to cross the CS/β-GP hydrogel structure prior to reach the medium, causing a slower but prolonged release kinetics^29^.

Finally, the effect of MSC-MVs on scavenging ROS was measured to assess the effectiveness of the paracrine role of MSCs loaded into CS/β-GP hydrogel. We cultured SH-SY5Y cells under oxidative stress conditions (induced by H_2_O_2_) in presence of supernatants collected from: (i) MSC alone; (ii) CS/β-GP hydrogel alone; (iii) MSCs + CS/β-GP hydrogel. As positive control (CTRL+), SH-SY5Y cells were also cultured in their specific medium and similarly stressed with H_2_O_2_.

Then the DCFHA assay was carried out comparing the different conditions. This assay measures the level of oxidative stress expressed as relative fluorescent unit (RFU). The results showed that both “MSCs”, “CS/β-GP” and “MSCs + CS/β-GP” were able to exert an anti-oxidative effect (Fig. 7G) as shown by a RFU reduction of 58.36% (MSCs), 37.81% (CS) and 65.77% (MSCs + CS) with respect to CTRL+ (cells in stressed condition). These data confirm that CS/β-GP does not affect the paracrine ability of MSCs, and also demonstrate that CS/β-GP alone is able to reduce the cellular oxidative stress.

### *In vivo* injection of MSCs-CS/β-GP hydrogel: a proof-of-concept study

**Figure 8:**
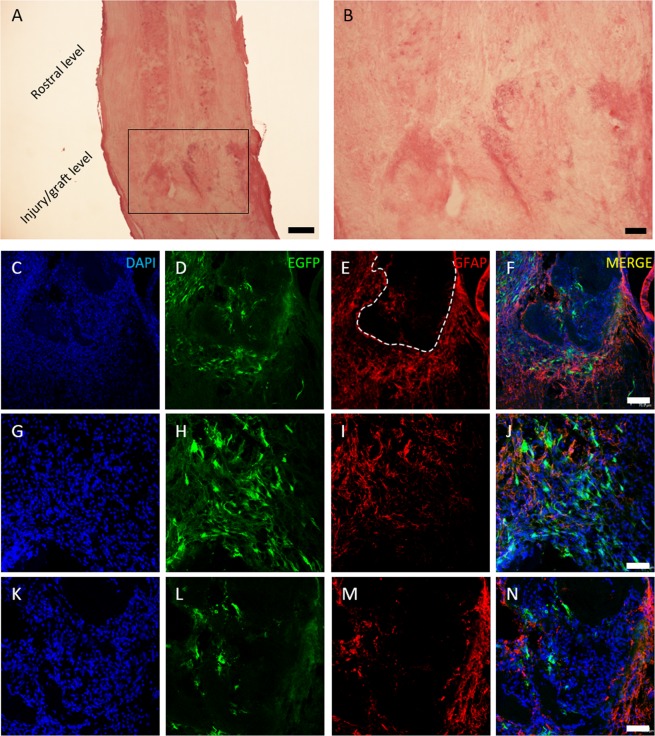
MSC and CS/β-GP hydrogel transplantation. Hematoxylin/eosin staining shows the uninjured rostral spinal level and the lesion/graft site (**A**); in (**B**) a higher magnification of the injury level. In blue DAPI nuclei (**C**-**G**-**K**), in green EGFP-positive MSCs (**D**-**H**-**L**), in red astrocytes forming the glial scar at the lesion site (**E**-**I**-**M**) and the three previous images overlapped (**F**-**J**-**N**). In (**C**–**F**) the lesion area at low magnification shows astrocyte distributed around the glial cyst (dashed line in **E**). At higher magnification MSCs are visible around (**H**) and inside the lesion (**L**). Scale bar: (**A**) 300 μm, (**B**) 100 μm, (**C**–**F**) 70 um, (**G**–**N**) 40 um.

In order to test *in vivo* the MSC survival in presence of CS/β-GP and to verify the hydrogel implant feasibility, 150,000 EGFP-positive MSCs were embedded in CS/β-GP hydrogel and injected immediately after murine SCI transection. After 1 week, the injury site was evident: as showed by hematoxylin/eosin staining (Fig. 8A,B), the complete spinal cord transection consistently interrupted the ascending/descending tracts. Numerous MSCs were visible at the lesion level. As shown in Fig. 8, MSCs were found both inside the lesion (as visible in D and, at higher magnification, in L) and surrounding it (H). Moreover, the astrogliosis (E, I, M) observed around the lesion is comparable to our previous works^30,31^ confirming that the addition of CS-based cell carrier did not affect the scar formation.

## Discussion

In the injured spinal cord, the beneficial effect of MSCs on tissue repair and inflammatory modulation have been extensively described^32^ and mainly ascribed to paracrine mechanisms. MSCs secrete a wide array of growth factors, chemokines, cytokines and extracellular vesicles (commonly referred to as secretome), acting as carriers of signal molecules regulating cell-to-cell and cell-extracellular matrix communications^33,34^. Authors demonstrated that MSCs transplanted into the injured murine spinal cord were able to reduce the lesion volume and improve the functional recovery^30^ thanks to the release of MSCs secretome containing neurotrophic/anti-inflammatory cues. However, the number of survived MSCs is generally low, suggesting that the majority of transplanted cells die after implantation, probably due to the hostile environment in the spinal cord injury site^35^. The poor engraftment of the transplanted cells could be boosted by improving stem cell survival and changing the injured microenvironment.

In such scenario, the aim of this study was to explore the potential of novel engineered cell-based therapies to treat SCI. To this aim, we combined CS-based materials with MSCs in order (i) to ameliorate the injured microenvironment and maximize stem cell survival, (ii) to provide an efficient *in situ* release of bioactive molecules, including anti-inflammatory cytokines and growth factors, (iii) to support the healing process modulating the inflammatory reaction and reducing the glial scar formation and (iv) to create a favourable environment to axon growth and neuroregeneration.

Indeed, despite the promising results reported for the use of MSCs in traumatic injuries, numerous challenges still remain to be faced including modalities of transplantation, transplanted cell survival rate and uncontrolled transplanted cell migration^34,36^. To overcome the fundamental challenge of successful cell transplantation to the injured site while maintaining cell viability, the use of biomaterial-based cell carriers has been proposed and encouraging pre-clinical data were reported for the treatment of several pathologies such as pericapsular fibrotic overgrowth^37^, myocardial infarction^38^ and liver fibrosis^39^. Several polymers can be applied for the fabrication of injectable polymeric cell carries^40,41^ as well as CS-based materials while its application in tissue engineering is strongly encouraged by the evidence of its high biocompatibility, its ability to enhance cells growth and its intrinsic anti-inflammatory and anti-oxidant behavior^17,42,43^. Furthermore, the neuroregeneration potential of CS-based bio-constructs has been recently reported^44^. Briefly, CS substrates were able to induce the differentiation of cortical and spinal cord progenitor cells into cortical neurons and motor neurons as well as to promote the re-arrangement of synaptic active networks. Moreover, compared to rigid/semi-rigid scaffolds, hydrogels present a high-water content and a viscoelasticity level close to that of living tissues, simulating the spinal cord mechanical properties^45^. Although the usage of CS as former biomaterial of 3D rigid scaffold for spinal cord regeneration was reported^7,44^, its use as injectable cell-laden hydrogel to treat injured spinal cord was not been reported yet. Indeed, CS appears particularly suitable for tissue engineering thanks to its biocompatibility, non-toxicity and biodegradability, with a molecular structure similar to the one of glycosaminoglycans, the main polysaccharides components of the ECM^42,46^. Moreover, Chenite *et al*., demonstrated that by the addition of glycerophosphate, CS solution becomes thermo-sensitive at physiological pH^47^. Other studies, as the ones performed by Supper *et al*., demonstrated that CS- polyol-phosphate salts (PP) solutions can be obtained at physiological pH showing a transition from solution to gel which underwent upon temperature increase^21^. The gelation mechanism of the CS-PP thermo-sensitive gelling system is still not completely understood, however in 2013 Supper *et al*.^48^ characterized in depth this complex system and described its gelation mechanism. Here, we proposed a modified protocol to obtain the CS-PP solution using the β-GP as gelling agents. A physiological environment suitable for cell housing was obtained by a proper balance in the solution components (Table 1). Furthermore, the mini-invasive approach of the cell-based treatment was ensured by the injectability of the CS/β-GP solution at 4 °C which underwent a phase transition when injected into solution at physiological temperature (Fig. S3). Rheological analysis confirmed the fast gelation of the CS/β-GP solution at physiological temperature (Fig. 3).

The high permeability of the CS/β-GP hydrogel (Fig. 5) combined with the high porosity (Fig. 1) and its prolonged stability in aqueous environment (Fig. 4) are key features for successful cell *in situ* engraftment and cell survival^49^ as highlighted by the MSCs survival within the developed CS/β-GP hydrogel. The viability of encapsulating MSCs was demonstrated by analysing cell survival (Fig. 7A,B) as well as by confirming MSCs functionality maintenance namely their ability to release MVs which are appointed for neuroprotection and neuroregeneration^34,36^ (Fig. 7C–F).

Moreover, a reduction of ROS level due to the scavenging effect of MSC-released bioactive molecules was observed both in MSCs alone and encapsulated into CS/β-GP hydrogel (Fig. 7G). High levels of ROS are detected after tissue injuries resulting in ROS-mediated cell death^50^ which negatively effects the healing process. Interestingly, a ROS scavenging activities was measured for CS/β-GP hydrogel as previously reported by Je *et al*.^51^.

Finally, on the basis of the encouraging results obtained *in vitro*, we tested the CS/β-GP hydrogel loading MSCs in a murine SCI model for the first time, in order to preliminarily evaluate the cell survival *in vivo* and to test the technical feasibility of CS/β-GP injection (Fig. 8). Preliminary *in vivo* tests demonstrated a high MSC survival at the injured site 1 week after implantation and, based on our experience, the number of survived MSCs appeared higher than in our previous works^30,31^. MSCs were detected both around and inside the lesion, suggesting that the CS/β-GP hydrogel did not affect their normal cellular behaviour and probably helped a better integration into the host tissue.

In addition, CS/β-GP hydrogel did not foster astrogliosis at the injury level. Indeed, at 7 days after the hydrogel injection, the immune response/inflammation process in proximity to the lesioned area was qualitatively comparable to that observed in previous experiments^30^, indicating the good biointegration of the developed formulation. This is an important finding as a strong immune reaction is one of the most frequent effects triggered by implanted synthetic and natural scaffolds^52^.

## Materials and Methods

### Chitosan hydrogel preparation

CS was purchased from Heppe Medical Chitosan (Halle, Germany) having a deacetylation degree higher than 92.6% and Mw from 100 kDa to 250 kDa determined by gel permeation chromatography (coded as CS 95/100).

CS hydrogels were prepared starting from a 3.6% (w/V) CS solution in 0.2 M HCl (Sigma Aldrich, Milan, Italy). The starting CS concentration was selected as the maximum amount of CS which can be dissolved in a 0.2 M HCl solution and it was obtained by stirring the CS powder in 0.2 M HCl for 24 hours and stored at 4 °C until CS hydrogels fabrication. β-glycerophosphate disodium salt hydrate (β-GP - M_w_ = 306.123 g/mol, Santa Cruz Biotechnology) was used as gelling agent and β-glycerophosphate disodium salt solutions were prepared in milliQ water and precooled to 4 °C for 15 min prior to use. The gelling agent was added drop-by-drop to CS solution under gentle magnetic stirring and cooling in an ice-bath. Then, the CS/β-GP solutions were further stirred for another 15 min and stored at 4 °C. The amount of β-GP and the final concentration of the CS/β-GP solution was varied to assess the influence of those parameters to the gelation time at 37 °C and the pH value.

The amount of β-GP was varied according to the R value which is defined as the ratio among β-GP moles and the moles of amino groups into the CS chain (1)

1 $${{\rm{mol}}}_{{\rm{\beta }} \mbox{-} {\rm{GP}}}={\rm{R}}\,\ast \,{{\rm{mol}}}_{{{\rm{NH}}}_{2}}$$

where ${{\rm{mol}}}_{{\rm{\beta }}-{\rm{GP}}}$ are β-GP moles and ${{\rm{mol}}}_{{{\rm{NH}}}_{2}}$ the theoretical moles of amino groups into the CS chains calculated considering the CS molecular weight and the deacetylation degree.

### CS hydrogels characterization

#### pH values of the CS/β-GP solutions

The pH value of the CS/β-GP solutions was measured using a pH-meter (Hanna Instruments) after solutions preparation and monitored during the gelation process.

#### Gelation time in physiological and storage conditions

CS/β-GP hydrogel gelation was studied by maintaining CS/β-GP solutions (0.5 mL in Eppendorf tube) at 37 °C (physiological condition) and analysing the gel or sol state each 30 s by tube inversion (*tube inversion method*). The sol condition was defined as ‘flow liquid sol’ while gel condition was considered for ‘no flow solid gel’^53^.

#### CS hydrogel morphology

The surface morphology of CS/β-GP hydrogels after freeze-drying (Scanvac-CoolSafe, freezing temperature −20 °C and 48 hours freeze-drying time) was observed by scanning electron microscopy (SEM LEO – 1430, Zeiss) using an accelerating voltage of 15 kV, a working distance of 10 mm and a Tungsten filament. Prior to analysis, samples were sputtered with a thin gold layer under a vacuum chamber. Pore dimension was quantified by analyzing five SEM images of fractured sections using an image software (ImageJ1.44 g).

#### Fourier transform infrared-attenuated total reflectance spectroscopy (FTIR-ATR)

Chemical characteristics of the CS hydrogels were evaluated by an attenuated total reflection Fourier transform infrared (FTIR-ATR) spectrophotometer (Perkin-Elmer). Spectra were obtained in the range of 4000–600 cm^−1^ with a resolution of 4 cm^−1^ and 16 scans. A diamond crystal and an angle of incidence of the contact beam of 45° were used. The spectra are reported after blank subtraction (spectrum without sample) and analysed using the Spectrum software.

#### Qualitative analysis of hydrogel injectability

The injectability of CS/β-GP solution was evaluated by using a volumetric pump (World Precision Instruments) equipped with a traditional 5 mL plastic syringe using 4 different needles having different internal diameters of 21 G (0.51 mm), 22 G (0.41 mm), 25 G (0.26 mm), 30 G (0.16 mm). Tests were carried out at four different flow rates (3, 5, 8 and 10 mL h^−1^) at 4 °C. To assess the capability of the CS solution to gel *in situ* at 37 °C, a drop of pink ink was added to the CS/β-GP solution and then coloured CS/β-GP solutions were injected into two beakers containing distilled water at 37 °C and 4 °C, respectively. The qualitative behaviour of the injected CS/β-GP solution was evaluated by visual inspection.

#### Rheological tests

Rheological tests on CS/β-GP solutions were performed by using a stress-controlled rheometer (MCR302, AntonPaar GmbH, Graz, Austria) equipped with 50 mm parallel plate geometry. For each test, the CS/β-GP solution was poured on the refrigerated lower plate (0 °C). Temperature control was guaranteed with a Peltier system while de-hydratation was prevented by a water trap. The effect shear strain amplitude on hydrogel was measured by the shear strain test at 37 °C (rotational oscillation 1 Hz, strain from 0.01% to 500%), while the frequency sweep test was performed at three different temperatures (20 °C, 30 °C and 37 °C) using a strain of 1% over angular frequencies (ω) from 0.1 to 100 rad/s. For the strain sweep test and the frequency sweep test CS/β-GP solutions were heated at the chosen temperature and maintained in quiescent conditions for 30 minutes prior to test.

The time sweep test and the temperature sweep test were performed to measure the time and the temperature of CS/β-GP solution gelation, respectively. For both tests, rotational oscillation was set at a frequency of 1 Hz and a shear strain amplitude of 1% was applied. For the time sweep test the temperature was set at 37 °C immediately after starting the test to recapitulate the temperature shift during injection into the physiological environment while for the temperature sweep test the range of temperature was from 0 °C to 50 °C with an increasing rate of 1.5 °C/min. Rheological tests were performed in triplicate.

#### Equilibrium water content and stability tests

Swelling and stability tests were carried out on CS/β-GP hydrogels. 0.8 ml of CS/β-GP solution was poured into plastic vials (diameter 18 mm), incubated at 37 °C for 30 minutes. Then, 1.6 ml of phosphate buffer saline solution (PBS) pre-warmed at 37 °C was added into each vial and incubated at 37 °C. At defined time points (1 hour, 3 hours, 6 hours, 1 day, 2 days, 5 days, 7 days, 14 days e 28 days), PBS was removed and samples were weighted before and after freeze-drying (W_wet_i_ and W_dry_i_, respectively). The Equilibrium Water Content (EWC) was calculated as (2)

2 $$\mathrm{EWC}( \% )=\frac{{{\rm{W}}}_{{\rm{wet}} \mbox{-} {\rm{i}}}-{{\rm{W}}}_{{\rm{dry}} \mbox{-} {\rm{i}}}}{{{\rm{W}}}_{{\rm{wet}} \mbox{-} {\rm{i}}}}\ast 100$$

while the weight loss (WL) was calculated as (3)

3 $$\mathrm{WL}( \% )=\frac{{{\rm{W}}}_{{\rm{dryTeor}}}-{{\rm{W}}}_{{\rm{dry}} \mbox{-} {\rm{i}}}}{{{\rm{W}}}_{{\rm{dryTeor}}}}\ast 100$$

where W_dryTeor_ is the theoretical weight of the dried sample at t = 0.

At the different time points, the pH of the removed PBS was measured and Ninhydrin (2,2-dihydroxyindane-1,3-dione) assay (Sigma Aldrich) was performed on removed PBS to analyze the dissolution of CS by quantifying the presence of primary amino groups. Briefly, 100 µm of removed PBS were poured into a 96 wells-plate and then Ninhydrin assay reagents were added following manufacturer’s instructions. Absorbance at 570 nm (A570) was recorded using a plate reader (Victor X3, Perkin Elmer) and CS concentration was determined by comparison of the A570 to CS solution of known concentrations.

#### Permeability

The transport of nutrients through CS/β-GP hydrogels was determined using two fluorescein isothiocyanate−dextrans (FD, Sigma-Aldrich, Milano, Italy) having a Mw 3 kDa (FD-4) and a Mw 20 kDa (FD-20) which are generally used as models to assess the permeability to nutrients and small proteins, respectively^54^. CS/β-GP hydrogels were prepared pouring 0.8 ml of the CS/β-GP solution into plastic vials (diameter 18 mm) and incubated at 37 °C for 30 minutes. Then, 1.6 ml of PBS loading 1 mg/ml of FD-4 or FD-20 were added to each CS/β-GP hydrogel. At defined time points (1 hour, 3 hours, 6 hours, 1 day) the residual FD-4 or FD-20 content in the solution incubated with each CS/β-GP hydrogel was quantified by UV–visible plate reader (Victor X3, Perkin Elmer) at 490 nm and three samples were used for each time points. The amount of adsorbed FD-4 and FD-20 was defined as the difference between the initial and the residual FD-4 or FD-20 amount. At the same time points the change in colour of the CS/β-GP hydrogel due to FD-4 or FD-20 adsorption was monitored by visual inspection.

The diffusion coefficient of FD-4 and FD-20 through the CS/β-GP hydrogels was estimated as previously reported by Boffito *et al*.^53^ (4):

4 $$\frac{{w}_{t}}{{w}_{\infty }}=2\,{(\frac{Dt}{\pi {l}^{2}})}^{1/2}$$

where w_t_ and w_∞_ are the amounts of FD-4 or FD-20 at time t and initially in the PBS solution (respectively), l is the height of the PBS solution in contact with the CS/β-GP hydrogel and t is the time point. The diffusion coefficients were calculated as the slope of the plot w_t_/w_∞_ against t^1/2^ in the linear region.

### *In vitro* experiments

#### Experimental animals

C57BL/6J male mice for experimental procedures (cell culture and *in vivo* experiments) were purchased from Envigo (Udine, Italia), while BCF1 mice that express enhanced green fluorescent protein (EGFP) under the β-actin promoter were obtained by Dr. M. Okabe (Osaka University, Suita, Japan). Animals were maintained under standard conditions with free access on food and water. All experimental procedures on live animals were performed in accordance with national (Law 116/92 on Care and Protection of living animals undergoing experimental or other scientific procedures; permit number 17/2010-B, June 30, 2010) and European Community Council guidelines (Council Directive 86/609/EEC). Additionally, an ad hoc Ethical Committee of the University of Turin specifically approved this study. The number of animals and suffering caused were kept to a minimum.

#### MSC isolation and culture

Two month-old C57BL/6J or EGFP-positive mice were anesthetized with 3% isoflurane vaporized in O_2_/N_2_O 50:50 and sacrificed by cervical dislocation. Tibias and femurs were extracted, muscles and connective tissues removed and then cells were aspirated from bone marrow through a 22-gauge needle. Cells were centrifuged 5 minutes at 1,000 rpm in Minimum Essential Eagle Alpha Modification medium (MEM; Sigma-Aldrich), supplemented with 100 U/ml penicillin (Invitrogen-Gibco), 100 µg /ml streptomycin (Invitrogen-Gibco) and 2 mM of glutamine (Invitrogen-Gibco). Polystyrene dishes of 19.5 cm^2^ (BD Biosciences, Milan, Italy) coated with fetal bovine serum (FBS, Sigma-Aldrich) were used to plate 700,000 cells/cm^2^. MSCs were grown in the previously mentioned medium (MSC medium) supplemented with 10% of FBS, in a 37 °C incubator with 95% of humidity and 5% of CO_2_. After four days, the medium was replaced to remove floating cells and then replenished every 2–3 days. Ten days after plating, adherent cells were detached by trypsinization (tripsin, Invitrogen) and CD11b positive granulocytic cells removed by beads sorting: briefly, microbeads conjugated to monoclonal rat anti-mouse/human CD11b antibody (Miltenyi Biotec GmbH, Gladbach, Germany) were incubated with cells and loaded onto a MACS column (Miltenyi Biotec GmbH). CD11b-negative cells were retrieved and re-plated in their medium as described above.

#### SH-SY5Y culture

For some experiments, SH-SY5Y neuroblastoma cell line, kindly provided by Prof. M.G. Spillantini (Cambridge University), was used. The cells were cultured in Roswell Park Memorial institute (RPMI) 1640 medium (Gibco, Invitrogen) with 2 mM of glutamine (Invitrogen-Gibco) [supplemented with 10% FBS, 100 U/ml penicillin (Invitrogen-Gibco) and 100 µg/ml streptomycin (Invitrogen-Gibco)].

#### Qualitative observations of cell viability in presence of hydrogel

SH-SY5Y cells and EGFP + MSCs were plated inside or near a 100 µl drop of CS/β-GP hydrogel to qualitatively evaluate the effect of the hydrogel on encapsulated cells or on cells surrounding the hydrogels, respectively. A concentration of 20,000 cells was used and cells were incubated at 37 °C with 95% of humidity and 5% of CO_2_. Control cells (in absence of CS/β-GP hydrogel) were cultured in the same conditions and plated at the same concentrations. For cell encapsulated into the CS hydrogel, cell suspension was maintained at room temperature and then, added to the cooled CS hydrogel, gently mixed and plated into a Petri dish to form the 100 µl drops. The cell loading drops were immediately incubated at 37 °C with 95% of humidity and 5% of CO_2_ and media was added after 10 minutes to cover the drops.

The cells were cultured in these conditions for 7 days *in vitro* (7 DIV). Before performing immunofluorescence reactions, cells were washed with PBS 1X, fixed in PFA 4% for 20 minutes at RT and washed twice. The cells were examined using an inverted microscope (Nikon Eclipse TE 2000-U). As an alternative, we embedded the whole hydrogel drop (CS/β-GP and cells) in the cryostat medium (Killik, Bio-Optica, Milan, Italy) and, by using the cryostat (Microm HM 550), we cut the samples into 50 µm-thick sections to better visualize cells into the hydrogel.

For immunofluorescence, after 10 minutes in PBS-triton 0.25% and 30 minutes in blocking solution [0.25% PBS-triton and 10% normal donkey serum (NDS; Sigma-Aldrich)-pH 7.4], SH-SY5Y cells were incubated with 1:200 monoclonal mouse anti-nestin (Chemicon). Then the cells were washed in PBS 1X and incubated with the secondary antibody for 1 h at RT (Jackson Immuno Research Laboratories; donkey anti-mouse1:200 cyanine 3-coniugated). Finally the cells were incubated with 4′,6 Diamino-2 phenyindole Dilactate (DAPI; Sigma Aldrich) in PBS 1:50 for 3′.

The cell viability was verified by morphologically observing the cells plated near or inside the hydrogel. Pictures were taken on a Nikon Eclipse E800 light microscope.

### Cytotoxicity assay: 3-(4,5-dimethylthiazol-2-yl)-2,5-diphenyltetrazolium bromide (MTT) assay

The MTT assay was performed to evaluate CS/β-GP hydrogel cytotoxicity. MSCs were cultured in their medium in a 96 multiwell plate at a final concentration of 6,000 cells per well. When they reached the confluence, the culture medium was removed and substituted with: (i) MSC medium; (ii) the supernatant collected from CS/β-GP hydrogel (48 h contact; 0.1 g CS/β-GP per ml); (iii) MSC medium containing 30% dimethyl sulfoxide (DMSO, Sigma Aldrich). MSC medium was used as positive control (CTRL+), whereas MSC medium +30% DMSO was employed to induce complete cell death as negative control (CTRL−).

After 24 h incubation, the MTT assay was performed following Manufacturer’s Instructions (Sigma Aldrich). The supernatant was carefully removed and the MTT assay performed as follows: a volume of 100 µl of MTT solution (5 mg/ml in PBS) was added and the plate incubated at 37 °C for 3 h. Then, the MTT solution was discarded and 100 µl of DMSO were added and mixed to dissolve the formazan crystals. Absorbance was measured at 570 nm wavelength on a Multiskan EX (Life Technology). Six samples for each of the three conditions were analyzed. Cell viability was calculated as a percentage value compared to CTRL+.

#### Calcein-AM/ethidium homodimer-1 assay

To evaluate the viability of MSCs encapsulated into CS/β-GP hydrogel, we used the calcein-AM/ethidium homodimer-1 (Eth-1) assay (LIVE/DEAD® Cell Imaging Kit, Life Technologies, Thermo Scientific, USA): the staining can distinguish between live or dead cells (respectively positive for calcein-AM or Eth-1). MSCs were plated on 24 multiwell plate at a final concentration of 20,000 per well. The experiment was conducted analyzing two different conditions: i) MSCs grown inside CS/β-GP hydrogel (the cells were loaded into CS/β-GP prior to gelation exploiting the thermosensitive behavior of the developed CS/β-GP based solutions); ii) or MSCs grown alone as positive control. After 5 days, the medium was removed and cells washed once with PBS 1X at pH 7.4. According to the manufacturer’s protocol, 150 μl solution of 4 μM Eth-1 and 2 μM calcein-AM was added to PBS and the plate was incubated 30 minutes at 37 °C. The cells were then fixed in PFA 4% for 20 minutes and cell fluorescence was checked on a Nikon Eclipse 80i epifluorescence microscope equipped with a Nikon DS-Fi1 digital camera or with a Leica TCS SP5 confocal laser scanning microscope. For this experiment EGFP-negative MSCs (collected from C57BL/6j mice) have been employed.

#### Extracellular vescicles (EV) collection and analysis

In order to verify whether the presence of CS/β-GP hydrogel could alter the therapeutic efficacy of MSCs, we evaluated their MV secretion, by analysing their presence within the supernatant.

MSCs were cultured as described above and MV collection performed as described by Rad and coll., 2016^55^. Samples of three conditions (MSCs cultured in normal medium, MSCs cultured into CS/β-GP, and CS/β-GP alone) underwent three consequent centrifugations respectively followed by supernatant collection and transfer into the tubes: (1) 400 g for 10 minutes; (2) 2,000 g for 20 minutes; and (3) 10,000 g for 70 minutes, at 4 °C. Then the pellet was washed with PBS 1X and then centrifuged again at the same high speed to remove proteins. The pellet containing MVs was stored at −80 °C prior to being analysed.

The MV release was evaluated by dynamic light scattering (DLS; Zetasizer Nano S90, Malvern Instruments, Worcestershire, UK). Among extracellular vesicles, microvescicles are defined as vesicles having a size ranging from 100 nm to 1000 nm. MV size was calculated as the mean value of three measurements. The results graphically show the size distribution of MVs as intensity plots.

To further confirm the ability of MSCs loaded into CS/β-GP hydrogel to release MVs, a quart crystal microbalance with dissipation monitoring device (QCM-D) was used to detach the presence of MVs within supernatants. QCM-D measurements were performed with a QSense Explorer device equipped with an open module (Q-Sense, Sweden). Odd overtones (3, 5, 7, 9, 11, 13) were monitored to evaluate changes in frequency (Δf) and energy dissipation factor (ΔD) at crystal fundamental resonance frequency (5 MHz). Gold (Au) coated sensors (QSX301, Q-Sense, Sweden) and NHS-amine coupling coated sensors (QSX 341, Q-Sense, Sweden) were used and cleaned following manufacture’s instruction prior to use. Immediately after cleaning, an Au sensor was placed in the open module and 400 µl of 0.5 g/L polyethylenimine solution (PEI, Sigma Aldrich) were gently poured on the sensor surface using a micropipette to create a positive charge coating on the Au sensor. Then, PEI solution was removed, crystal surface was rinsed with PBS buffer and 400 µl of MV pellet suspended in PBS (collected as previously described) were poured on crystal surface. The surface of NHS-amine coupling sensors was functionalized using anti-CD81 and anti-CD9 (1:100, anti-CD81, EPR2949 ab92726; anti-CD9, EPR 4244 ab109201, Abcam, Milan, Italy) following manufacture’s protocol. Then, 400 µl of MVs pellet suspended in PBS (collected as previously described) were poured on crystal surface.

#### 2′,7′-Dichlorodihydrofluorescin diacetate (DCFH-DA) assay

The antioxidant ability of MSCs loaded into CS/β-GP hydrogel was preliminary assessed by quantifying the effect of released factors on reactive oxygen species (ROS) production, by DCFH-DA assay (Invitrogen).

Briefly, MSCs were cultured for three days in a 24 multiwell plate at the concentration 20,000/well, inside CS/β-GP or alone; in additional wells we also put CS/β-GP alone. SHSY-5Y cells were plated in a 24 multiwell plate at the concentration 7,000/well. After 3 days, half of SHSY-5Y medium was replaced with fresh medium and half of supernatant collected from: (i) MSCs alone; (ii) CS/β-GP alone; (iii) MSCs + CS/β-GP. SH-SY5Y cells cultured in their specific medium were used as CTRL+.

To induce an oxidative stress, after 24 h 400 µM of H_2_O_2_ were added into some wells and incubated at 37 °C for 1 h (the other wells were used as controls) (Fig. S1). To detect ROS, the supernatant was removed and substituted with 100 µM of DCFH-DA solution in serum-free DMEM for 30 min at 37 °C, following the manufacturer’s procedure. Then, once removed the solution, repeated freeze-thaw cycles were performed to lyse the cells, before reading the plate on a Multiskan EX (Thermo Scientific) at 485 nm (535 nm emission). The results were analyzed by one-way ANOVA and expressed as relative fluorescent units (RFU).

### *In vivo* Experiments

#### Surgery and cell-hydrogel implantation

To verify the implantation feasibility of CS/β-GP hydrogel *in vivo*, some animals underwent surgery (n = 3). Three months-old C57BL/6 J mice were deeply anaesthetized with 3% isoflurane vaporized in O_2_/N_2_O 50:50. The thoracic spine was exposed, the spinal muscles displaced laterally and a complete spinal cord transection was performed using a 27-gauge1/2 needle at T13 level. Immediately after the injury, the animals received directly into the lesion cavity a solution of 10 µl of EGFP + MSCs and CS/β-GP hydrogel (150,000 cells-culture medium 3 µl + CS/β-GP hydrogel 7 µl; outer tip micropipette diameter 100 µm). After the injection, the animals were sutured and the wound was disinfected.

#### Histological examination

One week after the lesion and cell-hydrogel injection, mice were deeply anaesthetized with 3% isoflurane vaporized in O_2_/N_2_O 50:50 and transcardially perfused with 0.1 M PB, pH 7.4, followed by 4% PFA in the same PB. The spinal cord (T10-L2 segment) was dissected and post-fixed for 2 h at 4 °C in the same perfusion fixative. Samples were transferred overnight into 30% in 0.1 M PB at 4 °C, embedded in cryostat medium (Killik) and cut on the cryostat (Microm HM 550) in longitudinal 50 µm sections.

To assess the spinal cord injury/transplantation area, some sections were stained with hematoxylin/eosin, dehydrated in graded ethanol baths (95–100%) and cleared in xylene. The samples were then observed by a Nikon Eclipse 80i epifluorescence microscope equipped with a Nikon DS-Fi1 digital camera.

For immunofluorescence, spinal cord sections were immunoreacted as follows. After 30 minutes in PBS-triton 2% and 1 h in blocking solution [0.2% Triton X-100 and 10% NDS (Sigma-Aldrich) in PBS pH 7.4], the sections were incubated in the same solution with 1:500 polyclonal rabbit anti-GFAP, at 4 °C overnight. Then the sections were washed in PBS 1X and incubated with the secondary antibody for 2 h at RT (Jackson Immuno Research Laboratories; donkey anti-rabbit 1:200 cyanine 3-coniugated).

For checking double staining and making 3D reconstructions, some samples were examined with a Leica TCS SP5 confocal laser scanning microscope. Photomicrographs were corrected for brightness and contrast with Photoshop CS2 software.

### Statistical analysis

Data are reported as average value ± standard deviation as experiments were repeated three times. GraphPad Prism® software was used for one or two way analysis of variance (ANOVA). Values *p < 0.05, **p < 0.01, ***p < 0.005, ****p < 0.001 were considered statistically significant.

## Conclusion

The combination of biomaterial scaffolds with stem cell therapies has been emerged as one of the more promising strategy to deal with unsolved medical issues such as spinal cord regeneration. The easy processability and its high biocompatibility make the CS-based hydrogel developed within this work a potential candidate as carrier for MSCs-based therapies. By exploiting the paracrine mechanism, the developed MSCs loading hydrogel could be applied in the treatment of several challenging diseases affecting the nervous system. Its biodegradability, immune tolerance and capability of maintaining an adequate physiological environment for cell survival makes this CS/β-GP hydrogel a promising candidate for further analysis to investigate the efficacy of the developed system in reducing glial scar formation through long-term *in vivo* tests.

## Supplementary information

Supplementary Information

## Data Availability

The datasets generated and/or analysed during the current study are available from the corresponding author on reasonable request. See Supplementary Information for the movies acquired within FEM analysis.
