# Supplementary material for: Chitosan-based hydrogel to support the paracrine activity of mesenchymal stem cells in spinal cord injury treatment
Source: Sci Rep. 2019 Apr 25;9:6402. doi: 10.1038/s41598-019-42848-w (PMC6483991; doi:10.1038/s41598-019-42848-w)
Supplement: Supplementary file 1 — Supplementary Information [file 41598_2019_42848_MOESM1_ESM.docx]

**Chitosan-based hydrogel to support the paracrine activity of mesenchymal stem cells in spinal cord injury treatment**

Boido M.^a^, Ghibaudi M.^a^, Gentile P.^b^, Favaro E.^c^, Fusaro R.^d^, Tonda-Turo C.^d^*

a Neuroscience Institute Cavalieri Ottolenghi, Dept. Of Neuroscience, University of Torino, Orbassano, 10043, Italy

b School of Engineering, Newcastle University, Newcastle Upon Tyne, NE1 7RU, United Kingdom

c Department of Medical Science, University of Turin, Torino, 10126, Italy

d Department of Mechanical and Aerospace Engineering - Polito^BIO^Med Lab, Politecnico of Torino, Torino, 10129 Italy

**SUPPORTING INFORMATION**


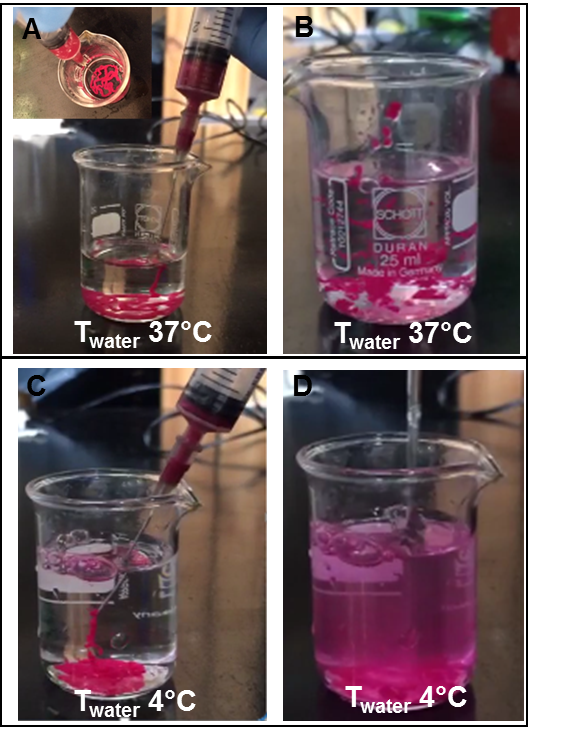


**Figure S1.** Representative images of CS/ β-GP solution injectability using a 25G needle. CS/ β-GP hydrogel was injected into water at 37°C (A) and 4°C (C). Then, the injected hydrogel was mechanically damage (B, D). When injected at 37°C the hydrogel did not colored the water confirming the formation of a stable gel (b). On the other hand, the colored water confirmed the instability of the CS/ β-GP solution at 4°C where no gelation occurred.

**
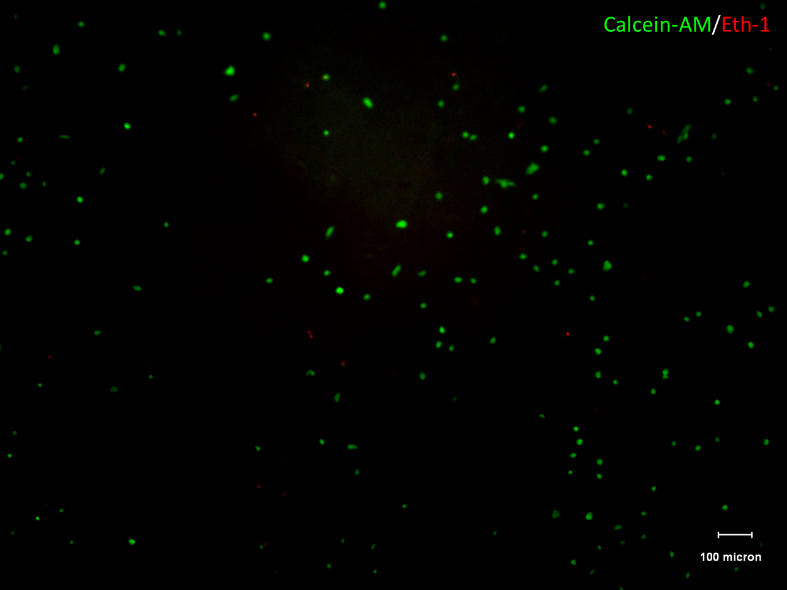
**

**Figure S2.** Calcein-AM/Eth-1 assay: rare MSC red-nuclei (Eth-1-positive) are detectable, whereas the green signal is intense and diffuse, demonstrating that the viability of MSCs is not negatively influenced by the presence of CS/β-GP hydrogel.


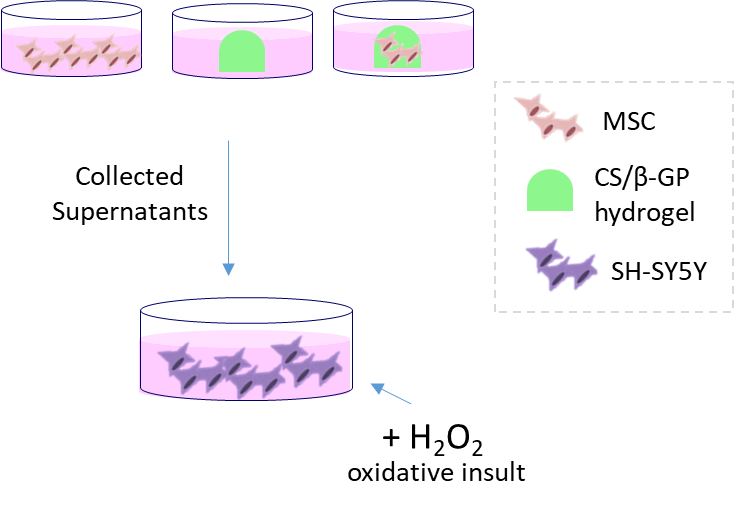


**Figure S3.** Schematic representation of experimental setup to test the paracrine role of MSCs loading into CS/β-GP hydrogel by quantifying the effect of released factors on reactive oxygen species production (ROS).
